# Identification of cellular microRNA-136 as a dual regulator of RIG-I-mediated innate immunity that antagonizes H5N1 IAV replication in A549 cells

**DOI:** 10.1038/srep14991

**Published:** 2015-10-09

**Authors:** Lianzhong Zhao, Jiping Zhu, Hongbo Zhou, Zongzheng Zhao, Zhong Zou, Xiaokun Liu, Xian Lin, Xue Zhang, Xuexia Deng, Ruifang Wang, Huanchun Chen, Meilin Jin

**Affiliations:** 1 State Key Laboratory of Agriculture Microbiology, Huazhong Agricultural University, Wuhan 430070, Hubei Province, P. R. China; 2 Laboratory of Animal Virology, College of Veterinary Medicine, Huazhong Agricultural University, Wuhan 430070, Hubei Province, P. R. China

## Abstract

H5N1 influenza A virus (IAV) causes severe respiratory diseases and high mortality rates in animals and humans. MicroRNAs are being increasingly studied to evaluate their potential as therapeutic entities to combat viral infection. However, mechanistic studies delineating the roles of microRNAs in regulating host-H5N1 virus interactions remain scarce. Here, we performed microRNA microarray analysis using A549 human lung epithelial cells infected with a highly pathogenic avian influenza virus. The microRNA expression profile of infected cells identified a small number of microRNAs being dysregulated upon H5N1 influenza A virus infection. Of the differentially expressed microRNAs, miR-136 was up-regulated 5-fold and exhibited potent antiviral activity *in vitro* against H5N1 influenza A virus, as well as vesicular stomatitis virus. On the one hand, 3′-untranslated region (UTR) reporter analysis revealed a miR-136 binding site in the 3′ UTR of IL-6. However, on the other hand, we subsequently determined that miR-136 meanwhile acts as an immune agonist of retinoic acid-inducible gene 1 (RIG-I), thereby causing IL-6 and IFN-β accumulation in A549 cells. Overall, this study implicates the dual role of miRNA-136 in the regulation of host antiviral innate immunity and suggests an important role for the microRNA-activated pathway in viral infection via pattern recognition receptors.

H5N1 influenza A virus causes highly infectious and acute respiratory diseases in avian and mammalian hosts, including humans. The case fatality rates of avian H5N1 IAV infection in humans can reach as high as 60%^1^. Therefore, an urgent need exists for developing effective prophylactic or therapeutic agents to help control the spread of potentially pandemic avian H5N1.

MicroRNAs (miRNAs) are a class of small non-coding RNA molecules that are expressed in the cells of multicellular organisms and modulate gene expression, predominantly by inducing mRNA degradation or inhibiting translation^2^. Cellular miRNAs participate extensively in regulating innate immunity and are functionally linked to numerous diseases, including diseases of viral origin. Multitudinous publications have revealed that miRNAs regulate innate immunity through imperfect complementarity with host gene transcripts. For instance, miR-146 had been shown to target key elements of the MyD88 signalling pathway, including interleukin-1 receptor-associated kinase 1 and TNF receptor-associated factor 6, and was recently identified as a regulator of enterovirus replication^3^,^4^. miR-155 directly targets the Fas-associated death domain protein and IKKε, leading to repressed NF-κB activation, and miR-155 expression also down-modulates inflammatory cytokine production in response to microbial stimuli^5^. miR-92a decreased the activation of the JNK/c-Jun pathway and the production of inflammatory cytokines in macrophages by targeting mRNA encoding the MKK4 kinase^6^. In addition to modulating the expression of host immune-associated genes, miRNAs also mediate antiviral defence mechanisms by targeting viral transcripts^7^,^8^,^9^. For example, a few miRNAs, such as miR323, miR491, miR654, and let-7c had been shown to restrict IAV replication by directly targeting viral gene segments of H1N1 strains^10^,^11^.

Furthermore, mounting evidence has demonstrated that endogenous miRNAs can function as ligands of toll-like receptors (TLRs) and other pattern recognition receptors (PRRs), such as retinoic acid inducible–gene 1 (RIG-I) and protein kinase R (PKR), leading to serial signalling activation. In previous studies, miR-21 and miR-29a are reported to bind to human TLR8 or murine TLR7, thereby increasing the secretion of the proinflammatory and prometastatic cytokines IL-6 and TNF-α^12^. Several other miRNAs are linked to TLR-mediated activation through exosomal pathway-dependent processes^13^. More recently, miR-145 was shown to induce immune responses through RIG-I recognition^14^. These findings encouraged investigators to identify potential immunostimulatory miRNAs that contribute to antiviral host responses or immunotherapy.

To provide insights into the extent of miRNA regulation induced by IAV infection, miRNA microarray analysis was performed in human lung epithelial cells (A549) exposed to A/duck/Hubei/hangmei01/2006 (designated as H5N1/HM). We identified several miRNAs that were responded to virus infection, including miR-136, which was selected for further detailed analysis. Collectively, miRNA-136 effectively antagonized H5N1 influenza A virus replication *in vitro* and mechanistic studies defined miR-136 as an IL-6 repressor, simultaneously as an immune trigger of RIG-I signalling, which implicated the pleiotropic and intricate effects of miR-136 in modulating immune activation during infection.

## Results

### miRNA expression profile analysis

To evaluate miRNA expression profiles during H5N1/HM infection, miRNA microarray analysis was performed as described in materials and methods section. The expression of most cellular miRNAs did not change significantly in response to infection, and seven differentially expressed miRNAs were identified using the cut-off criteria of absolute fold change ≥1.5 or ≤0.67 and P ≤ 0.05 (Supplementary Table 1).

**Figure 1:**
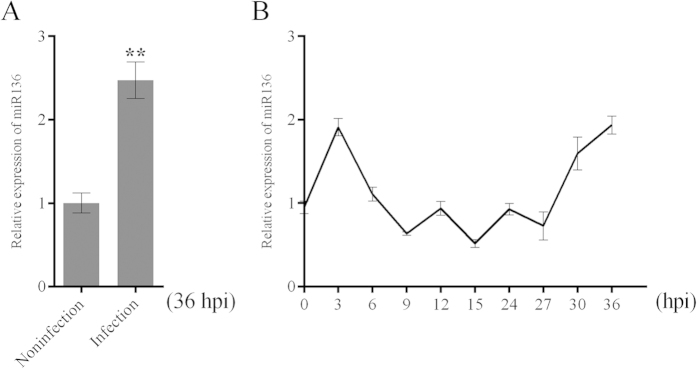
miR-136 expression analysis in H5N1/HM infected cells. (**A**) A549 cells were infected with H5N1/HM virus (MOI = 0.2) or mock infected for 36 hours, after which miR-136 expression levels were measured by RT-qPCR. (**B**) Relative expression levels of miR-136 were measured in virus-infected (MOI = 0.2) samples obtained at the indicated time points. Results are presented as mean ± SD of 3 independent experiments. **P < 0.001, as determined by performing a Student’s t test. hpi, hours post infection.

Here, we selected miR-136 for RT-qPCR assays, the results of which were consistent with our microarray findings in terms of miR-136 up-regulation and statistical significance (Fig. 1A). miR-136 expression was monitored following H5N1/HM infection in a time-dependent manner. As shown in Fig. 1B, oscillations in miR-136 expression levels were observed, suggesting the complexity of miRNA regulation disrupted by viral infection. These data indicated that miR-136 might be involved in virus-associated responses.

### Suppression of H5N1 and VSV replication in A549 cells by miR-136

**Figure 2:**
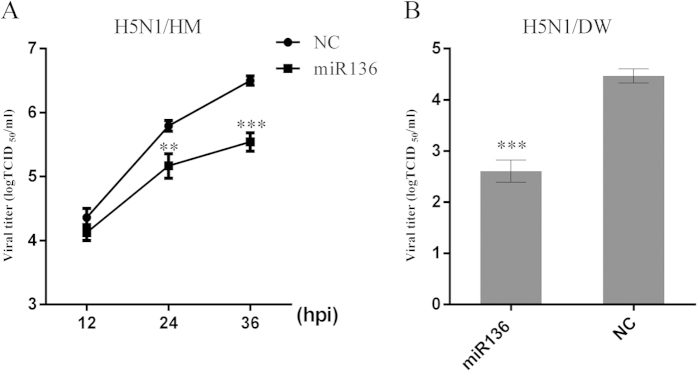
Overexpression of miR-136 inhibits H5N1 IAV in A549 cells. 12-well planted A549 cells were transfected with miR-136 or NC at final concentrations of 60 nM in culture supernatants. At 24 hours post-transfection, cells were infected with H5N1/HM or H5N1/DW at an MOI of 0.1 or 0.2, respectively. (**A**) H5N1/HM virus titres present in A549 culture supernatants were determined at the indicated time points by TCID_50_ determinations. (**B**) Virus titres in cultures supernatants were determined at 24 hours post-H5N1/DW infection. Viral titre values are presented as mean ± SD of 3 independent experiments. *P < 0.05, ***P < 0.001, as determined by performing a Student’s t test. hpi, hours post infection.

To investigate the effects of mature miR-136 on H5N1/HM replication, we preferentially determined that viral mRNAs were sharply decreased using a plasmid-based expression system of miR-136 (pCDNA3.1-miR136) (Supplementary Figure S1A, B). Furthermore, A549 cells were transfected with a chemosynthetic miR-136 mimic or the NC, followed by infection with H5N1/HM, using a multiplicity of infection (MOI) of 0.1. We then harvested cell culture supernatants at the indicated time points, and serially diluted them in Madin-Darby Canine Kidney (MDCK) cell cultures for TCID_50_ determinations. We found that the miR-136 significantly decreased viral titres at 24 and 36 hours post-infection, in comparison to the NC transfection (Fig. 2A). Next, we further determined whether miR136-mediated virus repression was specific to H5N1/HM. We selected another subtype H5N1 A/chicken/Hubei/327/2004 strain (designated as H5N1/DW; MOI = 0.2) for further evaluation. As shown in Fig. 2B, the titre of H5N1/DW was lowered by 1.8 logTCID_50_ at 24 ours post-infection in cells transfected with miR-136. These results indicated that miR-136 exerts potent antiviral activity against various H5N1 IAV strains.

Furthermore, we examined whether the antiviral activity of miR-136 was specific to IAV. Antiviral assays performed using a vesicular stomatitis virus (VSV) infection model. A549 cells were transfected with miR-136 or the NC, and cells were subsequently infected with a recombinant virus expressing enhanced green fluorescent protein (VSV-eGFP), using MOIs of 1 or 10. eGFP expression levels were detected by fluorescence microscopy and flow cytometry. Both the microphotographs and flow cytometric data showed that the miR-136 exhibited strong anti-VSV activity (Supplementary Figure S2), suggesting that miR-136 exerts a wide-spectrum of antiviral activity that is not limited to H5N1 IAV.

### miR-136 targets the IL-6 3′UTR, but enhances IL-6 expression

Because miR-136 restricted both IAV and VSV propagation, we therefore mainly focused on exploring host factors that may be involved in these antiviral processes. Initially, we sought potential miR-136 targets among host factors that may affect H5N1 IAV replication. Computational genome-wide predictions for miR-136 targets in 3′UTR regions were performed, using the miRanda target prediction algorithm in combination with the online TargetScan, PITA, and RNA22 programs^15^. Genes that were selected at least by 2 algorithms or were closely related to the IAV life cycle were selected for further analysis (Supplementary Table 3)^16^. However, only 14 of the candidate genes were validated by luciferase assays performed in this study (Supplementary Figure S3).

**Figure 3:**
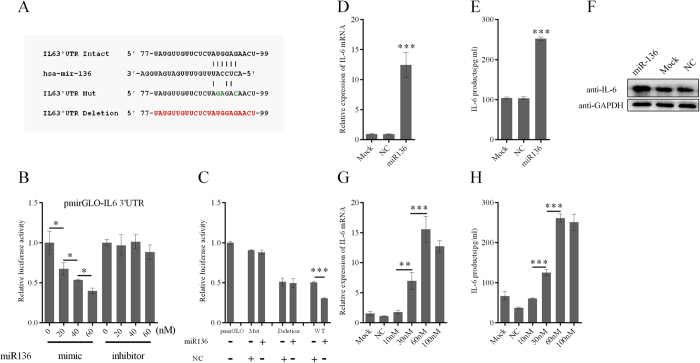
Effect of miR-136 on IL-6 expression in A549 cells. (**A**) Schematic representation of mutant IL-6 3′UTR reporter constructs. Red and blue letters indicate nucleotides that were deleted or point mutated, respectively. (**B**) A miR-136 or inhibitor (20 nM, 40 nM, or 60 nM) were cotransfected with the pMIR-IL-6 3′UTR reporter plasmid into 293T cells. Luciferase 0 nM mimic or inhibitor were assigned a value of 1 for normalization purposes. (**C**) 293T cells were cotransfected with the indicated oligonucleotides and either the parental pmirGLO vector, or a pmirGLO construct encoding wild type, point mutant, or deletion mutant variants of the IL-6 3′UTR. Luciferase activities were measured at 24 hours post-transfection, and the results shown reflect fold-inductions in luciferase activities relative to cells transfected with the empty pmirGLO vector. Cells were transfected with miR-136 or NC at a concentration of 60 nM. At 24 hours post-transfection, IL-6 mRNA levels (**D**) were detected by RT-qPCR, and IL-6 protein expression in cell supernatants (**E**) and sediments (**F**) were measured by ELISAs and western blots, respectively. Dose-dependent inductions of IL6 mRNA (**G**) and protein (**H**) expression by the miR-136 were detected using RT-qPCR and ELISAs, respectively. Results are presented as mean ± SD of 3 independent experiments. *P < 0.05, **P < 0.01, ***P < 0.001, as determined by one-way ANOVA with Bonferroni’s post hoc multiple comparison test (**B,D,E,G,H**) or Student’s t test (**C**). The blots were run under the same experimental conditions. Full-length blots/gels are presented in Supplementary Figure 5.

Repetitively, we found that miR-136 reduced firefly luciferase activity in cells transfected with reporter constructs with IL-6 3′UTR inserts (Fig. 3B). We also validated the miR-136 binding site in the IL-6 3′UTR by site-directed mutagenesis and deletion experiments (Fig. 3A). The miR-136 inhibited the luciferase activity of a reporter encoding an intact 3′UTR by 40%, compared to that observed following treatment with the NC. However, no inhibitory effect was observed in either the point-mutated IL-6 variants or the IL-6 deletion variants (Fig. 3C). These results suggested a miR-136 response element was present in the 3′UTR of IL-6. Based on these findings, we subsequently evaluated the regulation of miR-136 on endogenous IL-6 expression. To our great surprise, IL-6 mRNA expression level was elevated by 10 times with miR136 plasmid transfection (Supplementary Figure S4A). Thereafter, A549 cells were transfected with miR-136 mimic, the NC, or mock transfected. At 24 hours post-transfection, IL-6 mRNA was detected by RT-qPCR (Fig. 3D). IL-6 protein expression levels in culture supernatants and cell lysates were analysed by ELISAs and western blots, respectively (Fig. 3E,F). Dose-dependent effects of the miR-136 on IL-6 induction were observed (Fig. 3G,H). Together these results indicated that cellular IL-6 expression was greatly enhanced following transfection with either plasmid-expressed miR-136 or miR-136 mimics.

**Figure 4:**
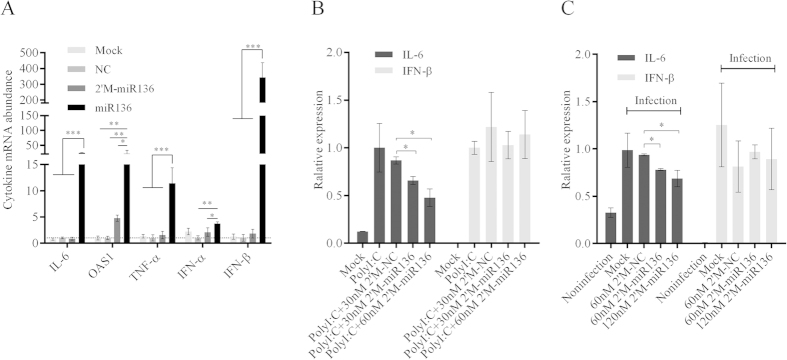
Cellular cytokines detection based on miR-136 transfection. (**A**) A549 cells were mock-transfected or transfected with NC, miR-136, or 2′-O-methyl modified miR-136 for 24 hours, using final mimic concentrations of 60 nM in the culture supernatants. Subsequently, the relative abundances of IL-6, OAS1, TNF-α, IFN-α, and IFN-β mRNAs were measured by RT-qPCR. (**B**) A549 cells were mock-transfected or transfected with 2′-O-methyl modified NC (60 nM), or 2′-O-methyl modified miR-136 (30 or 6  nM) for 12 hours, and then subjected to Poly (I:C) transfection (0.01 μg/well) with the exception of mock-transfection. At 12 hours post Poly (I:C) treatment, cellular IL-6 and IFN-β mRNAs were detected by RT-qPCR. (**C**) A549 cells were mock-transfected or transfected with 2′-O-methyl modified NC (60 nM), or 2′-O-methyl modified miR-136 (60 or 120 nM), and then infected with H5N1/HM virus at an MOI of 5. At 6 hours postinfection, cells were harvested and the expression of IL-6 and IFN-β was measured by RT-qPCR. Data are presented as mean ± SD from triplicate independent experiments. *P < 0.05, **P < 0.01, ***P < 0.001 as determined by one-way ANOVA with Bonferroni’s multiple comparison test. 2′M, 2′-O-methyl modified.

These results were consistent with results from a previous report showing that microRNAs are endogenous double-stranded RNAs (dsRNAs) that possess the ability to induce cytokine production^17^. To verify this possibility, the expression levels of additional cytokines were measured by RT-qPCR following miR-136 mimic or pCDNA3.1-miR136 transfection (Supplementary Figure S4B). IL-6, IFN-α, IFN-β, TNF-α, and OAS1 mRNA levels were all markedly up-regulated in group of naked miR-136 transfection as compared with 2′-O-Me-modified miR136 (Fig. 4A). It was worthy to note that 2′-O-Me-modified miR136 maintained slight cytokine induction with the exception of IL-6 mRNA level (though not significantly). This result was in agreement with previous conclusion by luciferase assays. To further consolidate our presumption, cells were transfected with 2′-O-Me-modified miR136 and modified NC, followed by Poly (I:C) stimulation. It demonstrated that 2′-O-Me-modified miR136 reduced IL-6 mRNA level by 25%-45% however had no significant effects on IFN-β expression (Fig. 4B). Thereafter, we also asked whether the miR-136 would contribute to IL-6 mRNA suppression during virus infection. We stimulated the 2′-O-Me-modified miR-136- or modified NC-transfected A549 cells using H5N1/HM virus at a MOI of 5 for 6 hours. As shown in Fig. 4C, we observed the 17%–25% inhibitory effect of 2′-O-Me-modified miR-136 on IL-6, but not on IFN-β, mRNA expression induced by H5N1/HM virus infection. Altogether, these findings suggest that while miR-136 targets IL-6 mRNA, it may simultaneously mediate the activation of innate immunity.

### miR-136 triggers innate immunity by acting as a ligand for RIG-I

**Figure 5:**
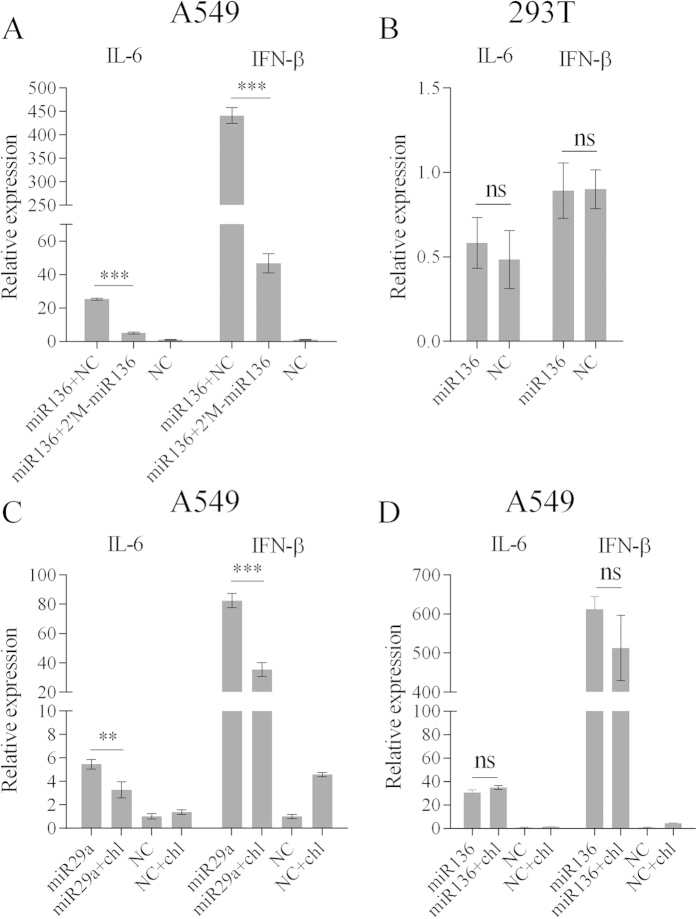
miR-136 activates innate immunity independent of TLRs pathway. (**A**) A549 cells were cotransfected with naked miR-136 and either NC or 2-O-methyl modified miR-136. At 24 hours following cotransfection, IL-6 and IFN-β mRNAs were detected. (**B**) Measurement of IL-6 and IFN-β mRNA expression levels in 293T cells exposed to miR-136. (**C,D**) Chloroquine (10 μM)-treated A549 cells were transfected with 60 nM miR-29a or miR-136, and IL-6 and IFN-β mRNA expression levels were determined by RT-qPCR. Data are presented as mean ± SD from triplicate independent experiments. **P < 0.01, ***P < 0.001, as determined by one-way ANOVA with Bonferroni’s multiple comparison test (**A,C,D**) or Student’s t test (**B**). 2′M, 2′-O-methyl modified; chl, chloroquine; ns, no significant.

Recently, several miRNAs have been recognized as ligands of TLRs or RIG-I, in turn contributing to immune enhancement. We noted that dsRNA-induced cytokine production can be abolished by a 2′-O-methyl modification within the internal sequences^18^. As above described, we used selectively 2′-O-methyl-modified miR-136 duplexes to investigate miR-136 recruitment. As expected, the oligonucleotide modification of miR-136 sequences disabled its immunostimulatory ability (Fig. 4A). Moreover, when A549 cells were cotransfected with unmodified miR-136 and modified duplexes, less cytokine production was observed, as compared to levels in cells co-transfected with unmodified miR-136 and the NC (Fig. 5A). When miR-136 was transfected into 293T cells (a cell line that is insensitive to innate immune activation^19^), IL-6 and IFN-β expression was minimally induced (Fig. 5B). These results implied that miR-136-mediated immune stimulation was most likely associated with a PRR-linked recognition pathway.

Cytoplasmic TLR3, TLR7, and TLR8, localizing to endosomal membranes, have been characterized as effective miRNA receptors, and this process is strictly dependent upon the endosomal pathway^20^. To determine whether endosomal TLRs were responsible for miR-136 recognition, we pretreated A549 cells with 10 μM chloroquine (an inhibitor of endosomal acidification), which was maintained in culture after miR-136 transfection. We used miR-29a as a positive control (Fig. 5C). We observed that chloroquine treatment did not reverse the cytokine-inducing ability of miR-136 (Fig. 5D). These observations suggested that an alternative pathway was required for miR-136-mediated immunostimulatory activation.

**Figure 6:**
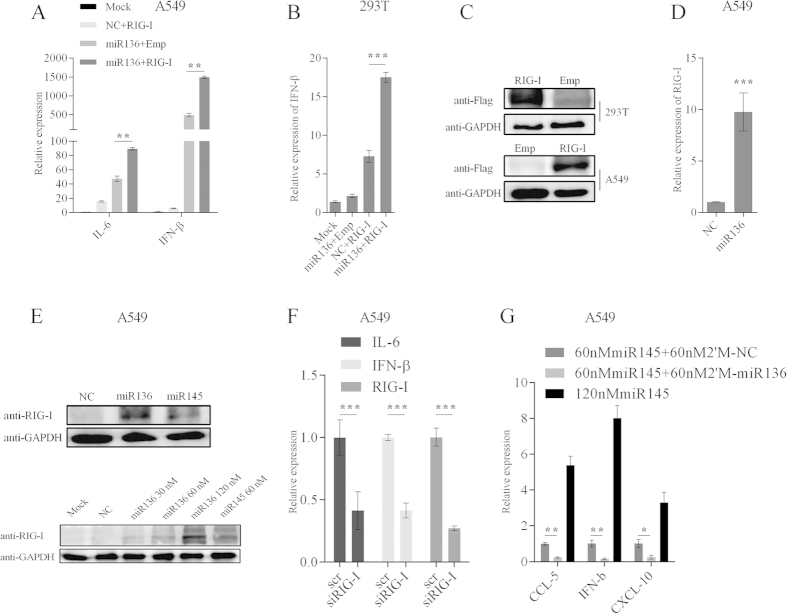
Identification of RIG-I as potential receptor for miR-136. (**A**) Expression of IL-6 and IFN-β was measured in A549 cells following cotransfection with the indicated RIG-I expression plasmids and either miR-136 or NC. (**B**) IFN-β mRNA expression levels were measured in 293T cells following the indicated treatments. (**C**) Validation of Flag-tagged RIG-I expression in 293T and A549 cells by western blotting. (**D**) Expression of RIG-I mRNA in A549 cells was determined following miR-136 transfection (60 nM, final concentration). (**E**) RIG-I protein levels in cells transfected with miR-136, miR-145, or NC were analysed by western blotting, using an anti-RIG-I antibody. (**F**) IL-6, IFN-β, and RIG-I mRNA expression levels after sequential 24-hour transfections, first with a siRNA targeting RIG-I (siRIG-I; 60 nM) or a scrambled siRNA, and then with 60 nM miR-136. (**G**) A549 cells were transfected for 24 hours with the indicated concentrations of miR-145, miR-145 mixed with 2′-O-methyl modified miR-136, or modified NC. Subsequently, mRNA expression of CCL5, CXCL10, and IFN-β were measured by RT-qPCR. The results are presented as mean ± SD of 3 independent experiments. *P < 0.05, **P < 0.01, ***P < 0.001, as determined by one-way ANOVA with Bonferroni’s multiple comparison test (**A,B,G**) or Student’s t test (**D,F**). Emp, empty vector; scr, scrambled siRNA; 2′M, 2′-O-methyl modified. The blots were run under the same experimental conditions. Full-length blots/gels are presented in Supplementary Figures 6, 7 and 8.

**Figure 7:**
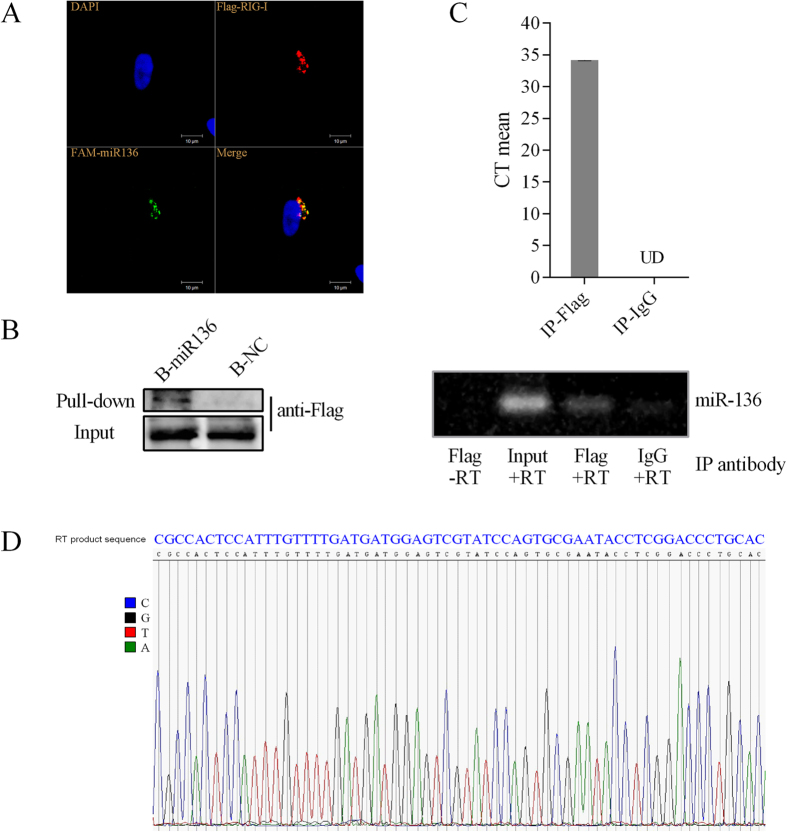
Cellular localization and association of miR-136 with RIG-I. (**A**) A549 cells on coverslips were transfected with FAM-labeled miR-136 (30 nM) and Flag-RIG-I plasmids (1 μg). 24 hours after transfection, cells were formaldehyde fixed and analysed by Immunofluorescence assay using antibody against Flag. Colocalization was analysed using Image J. (**B**) 293T cells in 75 cm^2^ flask were transfected with Flag-RIG-I plasmids (20 μg) for 36 hours. Total cellular protein was incubated with biotin-labeled miR-136 or negative control (8 μg), and subjected to streptavidin beads binding. Bound RIG-I protein was analysed by western blot. (**C**) RIP experiments were performed as described in methods section. The immunoprecipitated RNAs were subjected to RT-PCR assay for CT mean calculation and gel analysis. (**D**) The nucleotide sequences of RT-PCR product of miR-136 were verified by sequencing analysis. UD, Undetermined; B-miR136, biotin-miR136; B-NC, biotin-negative control. The blots/gels were run under the same experimental conditions. Full-length blots/gels are presented in Supplementary Figures 9, 10.

RIG-I is another established sensor for miRNA-induced innate immune responses. Thus, we next asked whether RIG-I was associated with miR-136 recognition. In A549 cells, overexpression of RIG-I and miR-136 significantly increased the abundances of IL-6 and IFN-β mRNAs, compared to the expression levels observed when cells overexpressed RIG-I or miR-136 alone (Fig. 6A,C). As shown in 293T cells, miR-136 clearly enhanced this efficacy on the basis of RIG-I transfection (Fig. 6B). Moreover, similar to previous finding^14^, we also detected up-regulation of RIG-I mRNA following miR-136 transfection (Fig. 6D). Western blot analysis showed a band of approximately 102 kDa in cells treated with miR-136 and positive control miR-145 (Fig. 6E upper), and this effect was enhanced by increasing miR-136 thansfection (Fig. 6E lower). This band was barely detectable in cells transfected with the NC. Together, these results suggested a close collaboration between RIG-I and miR-136. To further confirm this possibility, RIG-I specific siRNA was used to silence endogenous RIG-I expression. The knockdown efficacy of RIG-I expression reached nearly 70% according to RT-qPCR (Fig. 6F). After miR-136 delivery, IL-6 and IFN-β mRNA levels were down-regulated by approximately 60%, compared with scrambled siRNA transfectants (Fig. 6F). We further asked whether 2′-O-Me-modified miR136 could competitively inhibit cytokine production resulted by the known RIG-I ligand miR-145. As shown in Fig. 6G, miR-145 increased CCL5, CXCL10, and IFN-β expression in A549 cells. However, modified miR-136 significantly decreased their mRNA levels, in contrast with the 2′-O-Me-modified NC. We speculate that the modified miR-136 competitively bound to RIG-I, but failed to induce cytokine production, thus causing immunosuppression. If that is the case, this biological process could be closely associated with the recruitment of miR-136 to RIG-I. To confirm this assumption, confocal microscopy was used to examine for distribution following FAM-labeled miR-136 and FLAG-RIG-I cotransfection. Representative images are shown in Fig. 7A that miR-136 was shown to colocalizate with RIG-I protein in cytoplasm. On the basis of these results, we then performed RIP assay and miR-136 pull-down assay to further determine whether the protein-RNA complex was formed. The results indicated the physical interaction was true existed between miR-136 and RIG-I protein (Fig. 7B–D). Taken all these data together, it strongly suggests that RIG-I acted as a receptor for miR-136 recognition. Nevertheless, the precise mechanism involved in this biological process needs to be elucidated in our prospective studies.

### Endogenous miR-136 is associated with RIG-I-dependent signalling

**Figure 8:**
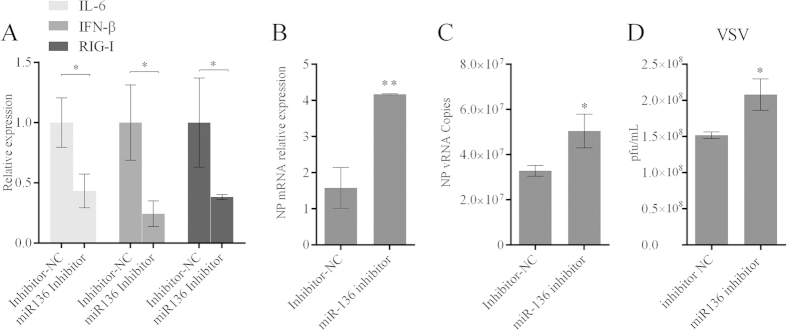
Neutralization of endogenous miR-136 negatively regulates RIG-I-dependent signalling. (**A**) 12-well planted A549 cells were subjected to miR-136 inhibitors or inhibitor negative control transfection using a concentration of 60 nM. 24 hours after transfection, cellular RIG-I, IFN-β and IL-6 mRNA levels were evaluated by RT-qPCR. (**B,C**) 12-well planted A549 cells were treated by the same procedure as above. 24 hours after transfection, cells were infected with H5N1/HM virus at an MOI of 0.5. At 36 hours postinfection, cells were collected and proceed with RNA extraction. NP mRNA abundance and vRNA numbers were evaluated by RT-qPCR assays. (**D**) 12-well planted A549 cells followed the same procedure as in (**A**). VSV was inoculated using an MOI of 0.01, and plaque assays were performed 24 hours later. The results are presented as mean ± SD of 3 independent experiments. *P < 0.05, **P < 0.01, as determined by performing a Student’s t test.

Since exogenous overexpression of miR-136 can activate RIG-I-dependent signalling pathway, we then investigated whether endogenous miR-136 are exactly involved in this biological process. The A549 cells were transfected with specific miR-136 inhibitors, and the mRNA levels of IL-6, IFN-β and RIG-I were determined 24 hours after transfection. It showed that blocking the function of miR-136 resulted in down-regulated RIG-I and its downstream genes IFN-β and IL-6 (Fig. 8A). Furthermore, we evaluated whether this impaired reduction of innate immunity influences the replication of H5N1/HM and VSV in A549 cells. Antiviral assays were performed, and RT-PCR results showed both the NP mRNA and vRNA levels of H5N1/HM virus in miR-136 inhibitor treated cells were considerably higher than those of the nonsense inhibitor control (Fig. 8B,C). Similarly, the inhibition of cellular miR-136 facilitates the propagation of VSV in A549 cells (Fig. 8D). These results indicated that endogenous miR-136 is identically closely associated with RIG-I mediated innate immunity.

## Discussion

MicroRNAs serve as multifunctional regulators and have been widely employed to study host-virus interactions. Many reports had shown that microRNAs are dysregulated in response to viral infection^21^,^22^. In this study, microRNA profiling also implied that microRNAs may serve potentially important roles in the infection process. Our data, together with a previous report^23^, prompted us to study the impact of miR-136 on the IAV life cycle. As expected, we observed potent antiviral activity against two subtypes of H5N1 IAV in miR-136-treated A549 cells. Recently, many antiviral miRNAs (miR-323, miR-491, miR-654, and Let-7c) have been found to reduce IAV replication by targeting the viral genome^10^,^11^. Furthermore, we also observed that VSV proliferation drastically decreased in the presence of miR-136, suggesting that miR-136 most likely interferes with IAV and VSV replication through a common mechanism.

To determine the role that miR-136 may play in response to viral infection, we were interested in exploring potential host factors that contribute to this process. Following traditional manner, several host factors were predicted and tested, and ultimately the IL-6 3′UTR was validated as a target of miR-136. However, on the other hand, cellular IL-6 mRNA and protein levels were dramatically up-regulated by miR-136 mimic or expression plasmids treatment. This inconsistency drew our attention to the fact that miR-136 is linked to cellular immune responses, which are not limited to sequence-specific targeting. We then found that serial cytokines including IL-6 and IFN-β were strikingly up-regulated under miR-136 transfection. Based on these results, we proposed that miR-136 mediated dual functions: post-transcriptional regulation and immune activation.

The mammalian innate immune response is critical for defending against pathogens. The involvement of miRNAs in this biological process had been highlighted in the last decade. Generally, miRNAs influence innate immunity in 2 well-studied ways: (і) as mentioned above, miRNAs can directly target immune-associated gene, which in turn suppresses or activates downstream signal pathways^24^, and (іі) sequence-specific siRNA or miRNAs may serve as ligands of PRRs and lead to immune activation. Known cellular responders for small dsRNAs mainly include TLR3, TLR7, and TLR8^12^,^13^,^25^,^26^,^27^, RIG-I^14^,^28^, and PKR^29^. The data presented in this report uncovered that miR-136 has high immunostimulatory efficiency and can be abrogated by chemical modification. This result was consistent with the conclusion that immunostimulatory dsRNAs with 2′-modified bases cause immunosuppressive effects^20^,^30^. Admittedly, not all dsRNAs or miRNAs promote immune activation. PolyU^28^ sequences or the GU^31^ motif were preferentially recognized by immune receptors. miR-136 contains a centrally located poly (UUUU) motif. In fact, our unpublished data have demonstrated that miR136 with poly (UUUU) mutation exhibited lower immunostimulation. Accordingly, it is conceivable that miR-136 most likely has triggered innate immunity just as miR21, miR29a or miR145 does. Our further studies support the hypothesis that miR-136-induced immune activation is at least dependent on RIG-I, but not on TLRs. Interestingly, we noted that miR-136, in A549 cells, had higher immunostimulatory effect in terms of IL-6 and IFN-β levels than that of known immunostimulatory dsRNA or miRNAs, such as miR21, miR29a, let7^32^, and RNA27^33^ (data not shown). All of these dsRNAs possess the immunostimulatory ability, however, the exact structural features of miR-136 that facilitate RIG-I activation and the involved mechanistic discrepancy are still of great interest.

In resistance to pathogens, RIG-I is known as a powerful sensory molecule in the innate immune system. Specialized motifs have been designed for RIG-I recruitment and can raise innate immunity in mice to combat IAV infection^34^. L. Martinez-Gil’s group took advantage of an RNA agonist of RIG-I as a viral vaccine adjuvant^35^. Similarly, miRNAs have potential for use as immune adjuvants and in immunotherapy. Hitherto, numerous concentrations have been focused on canonical miRNA-mRNA interaction, depending on which gene expression was regulated. Importantly, Fabbri *et al.*^27^ had emphasised the implication of miRNA-TLR or miRNA-PRR interaction on human pathology, which was not limited to cancer progress. In this study, miRNA136 was established as a novel endogenous RIG-I activator and may contribute to the control of virus diseases. Although, it is worth debate that physilogical level of miR-136 was too low to activate RIG-I signal pathway. However, neutralization of miR-136 with miR-136 inhibitor indeed reduced both cellular RIG-I and IFN-β mRNA level. Suppositionally, miR-136 might serve as a representative immune contributor among numerous microRNAs and potential immune activators in cells. In this regard, the contribution to immune activation and inflammatory repression by miR-136 should not be ignored, especially respond to viral invasion. As a hypothesis, combined with others’ observations, we speculate that cellular microRNAs may have profound biological relevance in maintaining or balancing cellular basal immune level.

In a previous report, miR-136 was identified as a negative regulator of Bcl-2 in glioma cells^36^. It is well known that the Bcl-2 gene is induced by activation of the IL-6 signalling pathway, and the Bcl-2 protein can be positively regulated on IL-6 stimulation. However, some types of immune-response genes are not expressed in glioma cells^37^, based on which cytokine expression was hardly affected by miR-136 overloading in Y. Yang’s studies. In a recent study, Sining Shen and his colleagues defined miR-136 as a new modulator in promoting Erk1/2 pathway activation by targeting PPP2R2A^38^. The Erk1/2 signal pathway belongs to mitogen-activated protein kinase signalling cascade that is activated by TLRs^39^. Our data, together with these findings, imply that miR-136 acts as a multifunctional regulator of crosstalk in signalling pathways and in different cell types. However, the specific mechanism whereby miR-136 modulates cellular signalling networks still needs to be further studied.

In summary, we observed that host cellular miRNAs were differentially expressed following H5N1 IAV infection. Although this study identified several miRNAs that may be closely involved in viral infection, additional experiments need to be performed to validate their biological functions. In the present study, we mainly characterize miR-136 as an IAV replication suppressor *in vitro*, the underlying mechanism of which is the enhancement of innate immunity by acting as a ligand of RIG-I. The collective data provide insight into the promising role of miRNAs for the treatment of IAV or other viral infections.

## Methods

### Cell culture

MDCK cells were obtained from the American Type Culture Collection (Manassas, VA) and propagated in Dulbecco’s Modified Eagle’s Medium (DMEM; Invitrogen, Carlsbad, CA), supplemented with 10% foetal bovine serum (FBS; GIBCO, Auckland, NZ). Human lung epithelial cells (A549) and human embryonic kidney cells (293T) were cultured in F12 and 1640 media (Invitrogen), respectively, which were supplemented with 2 mM L-glutamine, 10 mM HEPES, 1.5 g/L sodium bicarbonate, 4.5 g/L glucose, and 10% FBS.

### Viruses

The HPAI H5N1 virus strains, A/duck/Hubei/hangmei01/2006 (H5N1; designated as H5N1/HM) and A/chicken/Hubei/327/2004 (H5N1; designated as H5N1/DW), were isolated from a duck and a chicken, respectively. Viral titres in culture supernatants were measured by determining log10 TCID_50_/ml values in MDCK cells, as previously described^40^. All experiments with H5N1 viruses were performed in Animal Biosafety Level 3 laboratory. The recombinant vesicular stomatitis virus encoding enhanced green fluorescence protein (VSV-eGFP) was a gift from Harbin Veterinary Research Institute. eGFP expression levels directly reflect the degree of VSV replication. eGFP expression was visualized by fluorescence microscopy and analysed by flow cytometry. Virus plaque assays were carried out just following the procedures described in our previous study^41^.

### miRNA microarray analysis

A549 cells were cultured in T-flasks (25 cm^2^) in triplicate and identically processed by removing their growth medium, washed three times with PBS before adding serum-free medium containing H5N1/HM virus of 10^3^ TCID_50_ for 36 h. As control, another triplicate group of uninfected cells simulated the procedure for virus infection. At 36 hours post-infection, cells were washed thrice with PBS. The cells were treated with 1 ml TRIzol reagent (Invitrogen) and proceed with chloroform addition and phase separation. The aqueous phase was further subjected to RNA purification with the RNeasy Mini Kit (Qiagen, Valencia, CA), following the manufacturer’s instructions. miRNA microarray assays with extracted RNA samples involved labelling, hybridization, scanning, normalization, and data analysis steps, which were performed with Exiqon miRNA arrays (Vedbaek, Denmark), according to the manufacturer’s recommended protocols. Briefly, RNA qualities and concentrations were assessed by measuring optical density ratios at 260 nm/280 nm, using a NanoDrop spectrophotometer (NanoDrop Technologies, Wilmington, DE). RNA samples were labelled with the miRCURY™ Hy3™/Hy5™ Array Power Labelling Kit and hybridized with the miRCURY™ LNA (locked nucleic acid) Array (v.11.0). Each sample was processed in 3 independent hybridizations on chips, with each miRNA was spotted in quadruplicate. Labelling efficiencies were evaluated by detecting signals from control spike-in capture probes. LNA-modified capture probes corresponding to human mature sense miRNA sequences were spotted on slides. This set of LNA-modified probes had uniform melting temperatures of 72 °C against their complementary targets. Hybridizations between RNA samples and the spotted probes were performed with an Automated Hybridization Station (Lucidea Slide Processor; GE Healthcare, Piscataway, NJ). Signal scanning was performed with the Axon GenePix 4000B Microarray Scanner and raw image intensities were read with GenePix Pro software, V6.0. The signal intensity values obtained were normalized to per-chip median values and then applied to obtain geometric means and standard deviations for each miRNA.

All capture probes that have been developed for miRNAs in all organisms tested are indexed in the Sanger miRBase, Release 11.0 (http://microrna.sanger.ac.uk). More than 1700 human, mouse, and rat capture probes were included in the most recent version of the array (v.11.0), including all miRNAs annotated in miRBase 11.0, as well as all viral miRNAs related to these species. To evaluate the expression of miRNAs, the online R project analysis program (http://www.r-project.org/) was used to calculate correlation coefficients. The median of the signal intensity and local background values for each channel were used for correction. Values from uninfected A549 cells were used for normalization with the median normalization method. P < 0.05 was considered significant. The raw and normalized microRNA data have been deposited in NCBI’s Gene Expression Omnibus (GEO) database^42^ and are accessible through GEO Series accession number GSE61701.

### Vector construction and reagents

Chloroquine and Poly (I:C) were purchased from Sigma-Aldrich (St. Louis, MO). The Flag-RIG-I expression plasmid was generously provided by Zhengfan Jiang (University of Beijing). Genomic DNA encoding the hsa-mir-136 precursor was PCR-amplified from A549 cells and inserted into the pcDNA3.1+ vector (Invitrogen) between the XhoI and BamHI restriction sites. miR-136 targets were computationally predicted using the online miRanda software in combination with the online TargetScan, PITA, and RNA22 programs. The 3′UTRs of interest were PCR-amplified from genomic DNA and cloned into the luciferase reporter vector pmirGLO (Promega, Madison, WI). Mutant and deletion variants of the IL-6 3′UTR were constructed by PCR, using primers with built-in mutation sites. miR-136 mimic, negative control mimic (NC), miR-136 inhibitor, inhibitor control and other dsRNAs used in this study were obtained commercially from GenePharma (Shanghai, China). The accuracy of all DNA constructs was validated by sequencing. The sequences of the primers, siRNAs, and miRNAs used in this study are listed in Supplementary Table 2 or supplied upon request.

### Real-time quantitative PCR (RT-qPCR) assays

Total RNA was isolated using the TRIzol reagent according to manufacturer’s instructions. Genomic DNA was removed from RNA samples by incubation with DNase I, and 2 μg of total RNA was converted to cDNA using avian myeloblastosis virus reverse transcriptase (AMV; TaKaRa Biotechnology, DaLian, China) with an oligo-dT primer for mRNA and a special stem-loop primer for miRNAs^43^. Real-time quantitative PCR was performed on an ABI ViiA7 instrument (Applied Biosystems), using SYBR Green detection chemistry (Roche). Relative expression levels were calculated by applying the 2−^ΔΔCt^ method^44^ using reference gene GAPDH for mRNA and U6 snRNA for miRNA, and relative to the control samples.

To determine the number of copies of the H5N1/HM NP vRNA in miR-136 inhibitor treated cells, the cell total RNA was prepared using TRIzol reagent. And the viral RNA, from 2 μg of total RNA, was reverse transcribed with the Uni-12 primer. 4 μl of reverse transcribed products was subjected to real-time PCR analysis using the NP gene of the H5N1/HM virus construct plasmid at the standard concentration. The sequences of primers used for real-time quantitative PCR are contained in Supplementary Table 2 or available upon request.

### IL-6 ELISA and western blot analysis

Human IL-6 production in A549 cell supernatants was measured using a commercial human IL-6 ELISA kit (Dakeve, Shenzhen, China), following the manufacturer’s instructions. Western blot analysis was performed as previously described^45^ using antibodies against IL-6 (Abgent), Flag (Proteintech), RIG-I (Cell Signaling Technology), and GAPDH (Proteintech).

### Luciferase assays

293T cells were seeded into 24-well plates and transfections were performed using 6 μl of Lipofectamine 2000 (Invitrogen) mixed with 0.5 μg pmirGLO-3′UTR constructs and 1 μg pCDNA3.1–136 plasmids. In some experiments, 293T cells were also cotransfected with miR-136 or the NC, using final mimic concentrations of 60 nM. Cells were incubated for 24 hours and luciferase assays were performed using the Dual-Luciferase Reporter Assay System Kit (Promega), according to the manufacturer’s protocol. Relative firefly luciferase activities were normalized to Renilla luciferase activities.

### Immunofluorescence assay

To determine whether miR-136 were associated with RIG-I, 12-well planted A549 cells were co-transfected with a FAM-labeled miR-136 (30 M) (GenePharma) and FLAG-RIG-I expression plasmids (1 μg) for 24 hours. The cells were fixed with 4% paraformaldehyde, permeabilized using 0.1% Triton X-100 for 30 min. After washing, cells were incubated with primary antibody against FLAG for 1 hour, then secondary antibody for 40 min, washed and stained with DAPI (Invitrogen). Samples were examined by confocal microscopy with the LSM510 system (Carl Zeiss, Oberkochen, Germany). Colocalization was analysed using the software Image J (NIH).

### Biotin-labeled miR-136 pull-down assay

miR-136 pull-down assay was performed as previously reported with minor modifications^46^. 293T cells were planted in 75 cm^2^ cell culture flask, and Flag-RIG-I plasmids (20 μg) were transfected for 36 hours. Cell lysates were prepared and incubated with biotin-labeled miR-136 (GenePharma) or biotin-labeled negative control (8 μg) in 4 °C for 1 hour. The complexes were subjected to Streptavidin Dynabeads (Beijing 4A Biotech Company, Cat# MP003-05) pull-down for another 1 hour. Bound protein was analysed by western blot with anti-Flag antibody.

### RNA immunoprecipitation (RIP) assay

RIP assays were performed as previously described with minor modifications^47^. Generally, 293T cells were prepared in 75 cm^2^ cell culture flask. With ~80% confluence, cells were co-transfected with FLAG-RIG-I expression plasmids (15 μg) and miR-136 mimics (60 nM). After 36 hours transfection, cells were cross-linked with 1% formaldehyde for 15 min and washed twice with cold PBS. Cells were recovered by scraping and lysed in 1 ml RIP buffer (50 mM Tris-HCl PH = 7.4, 150 mM NaCl, 0.5% NP-40, 5 mM EDTA, 0.5 mM DTT, protease inhibitor PMSF, RNAse inhibitor (50 U/ml final)). Insoluble material was removed by centrifugation at maximum speed for 15 min at 4 °C. Cell extracts were quickly frozen at 80 °C. Fifty microliters of protein A/G beads were washed with PBS and then incubated with 5 μg FLAG antibodies or 5 μg IgG overnight with gentle rotation. The cell extracts were split into two fractions of 500 l each (for mock and IP) and added to the beads prepared above with gentle rotation for 4 hours at 4 °C. Beads were pelleted at 2,500 RPM for 30 s, the supernatant was removed, and beads were resuspended in 500 μl RIP buffer and repeated for twice, followed by one wash in PBS. The complex was eluted with 250 μl of elution buffer (1% SDS, 0.1 M NaHCO_3_). Crosslinking was reversed by adding NaCl to a final concentration of 200 mM and incubating at 65 °C for 3 hours. Protein was removed by incubating with proteinase K (20 μg; invitrogen) at 42 °C for 50 min, followed by Trizol extraction. The RNAs were treated with recombinant DNase I, followed by reverse transcription. Corresponding cDNA of miR-136 was subjected to qPCR analysis and semiquantitative reverse transcription-PCR. The PCR products were further cloned into pMD-18T vector for sequencing analysis.

### Statistical analysis

Statistical significances were determined using conventional Student’s t test or ANOVA with GraphPad Prism software (San Diego, CA). All the assays were run in triplicate and were representative of at least 3 independent experiments. Data are shown as means ± SD. A P value below 0.05 was considered statistically significant and the asterisks in all figures are defined, *p < 0.05, **p < 0.01, ***p < 0.001.

## Additional Information

**How to cite this article**: Zhao, L. *et al.* Identification of cellular microRNA-136 as a dual regulator of RIG-I-mediated innate immunity that antagonizes H5N1 IAV replication in A549 cells. *Sci. Rep.* **5**, 14991; doi: 10.1038/srep14991 (2015).

## Supplementary Material

Supplementary Information
