# Supplementary material for: Identification of cellular microRNA-136 as a dual regulator of RIG-I-mediated innate immunity that antagonizes H5N1 IAV replication in A549 cells
Source: Sci Rep. 2015 Oct 9;5:14991. doi: 10.1038/srep14991 (PMC4598873; doi:10.1038/srep14991)
Supplement: Supplementary Information [file srep14991-s1.pdf]

Identification of cellular microRNA-136 as a dual regulator of RIG-I-mediated innate immunity that antagonizes H5N1 IAV replication in A549 cells

Lianzhong Zhao, Jiping Zhu, Hongbo Zhou, Zongzheng Zhao, Zhong Zou, Xiaokun Liu, Xian Lin, Xue Zhang, Xuexia Deng, Ruifang Wang, Huanchun Chen and Meilin Jin

Supplementary materials

Supplementary Figure S1

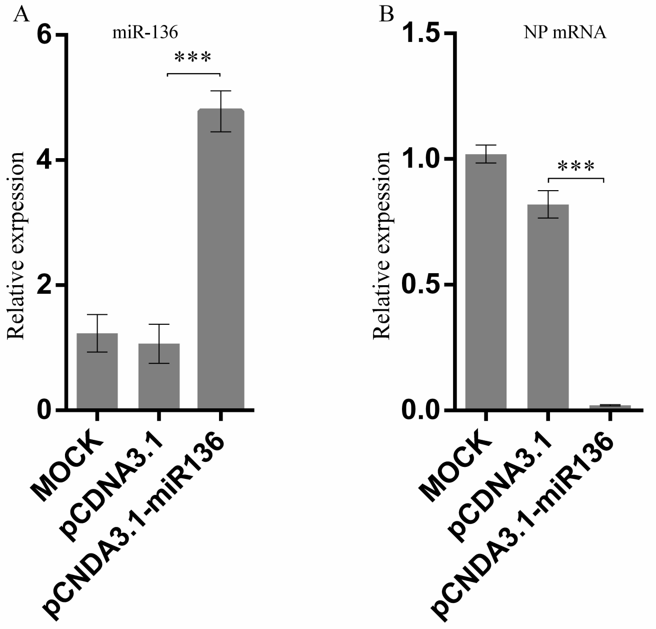

Supplementary Figure S1. Plasmid-based overexpression of miR136 reduced H5NA/HM NP mRNA abundance. (A) miR-136 expression level was detected in 12-well planted cells transfected with an amount of 1 μg pCDNA3.1-miR136, compared with that of pCDNA3.1 empty vector (1 μg) or mock transfection. (B) 12-well cells were transfected by the same procedure as above. After 24 hours transfection, cells then infected with H5N1/HM at an MOI of 0.2. Viral NP mRNA was evaluated by RT-qPCR at 24 hours post-infection. Results are presented as mean ± SD of 3 independent experiments. ***P < 0.001, as determined by one-way ANOVA with Bonferroni's multiple comparison test.

Supplementary Figure S2

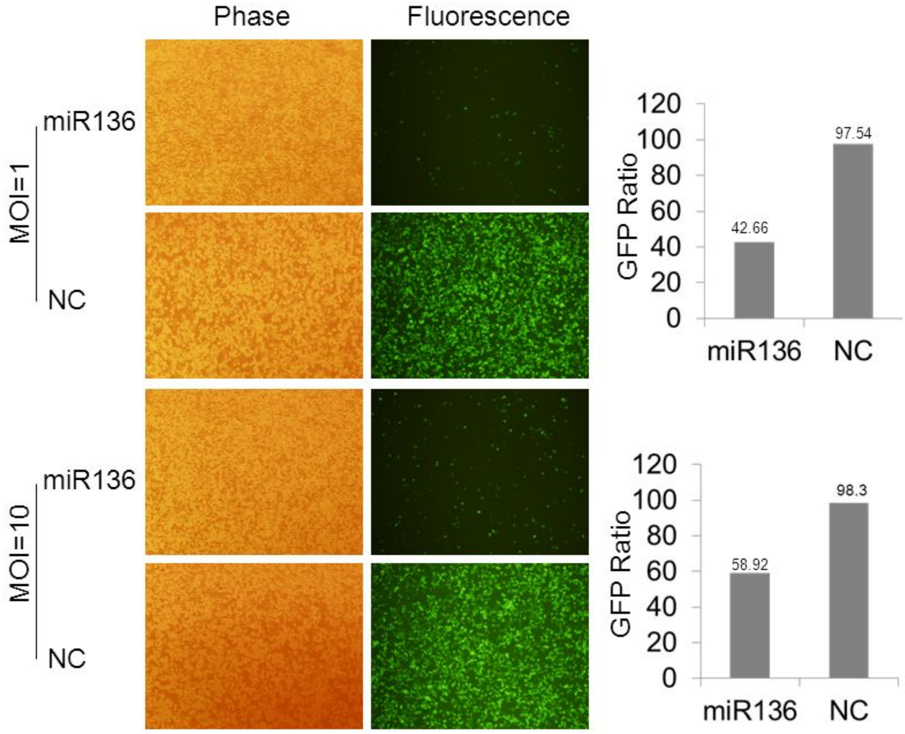

Supplementary Figure S2. A549 cells were transfected with a 60 nM concentration of miR-136 or NC. After a 24-hour transfection period, cells were infected for 24 hours with VSV-eGFP (MOI = 1 or 10). Microphotographs were taken, and the cells were analysed by flow cytometry to study GFP expression. The presented image is a representative result from three separate experiments.

Supplementary Figure S3

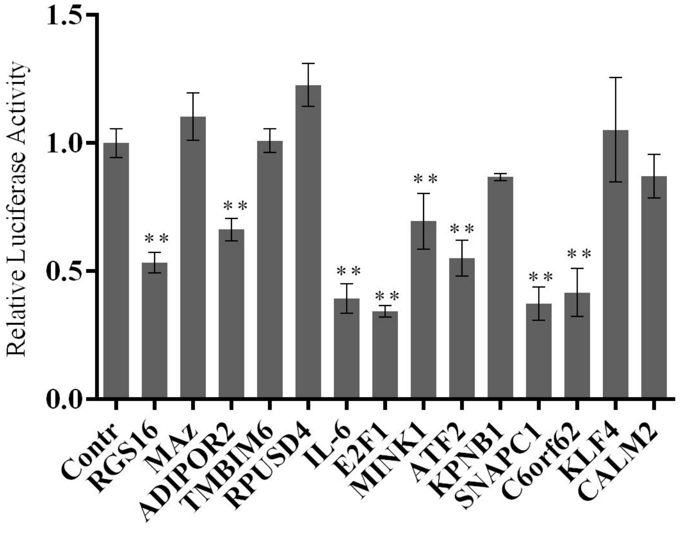

Supplementary Figure S3. miR-136 target analysis in 293T cells, using luciferase reporter assays. 293T cells were cotransfected with miR-136 and reporter constructs encoding the 3′UTRs of candidate genes, and luciferase reporter assays were performed at 24 hours post-transfection. The mean luciferase activity of control cells that were cotransfected with pCDNA3.1-miR136 and the empty reporter plasmid was set to a value of 1 for normalization purposes. Data are expressed as mean ± SD (n = 3). **P < 0.01, as determined by one-way ANOVA with Bonferroni's multiple comparison test.

Supplementary Figure S4

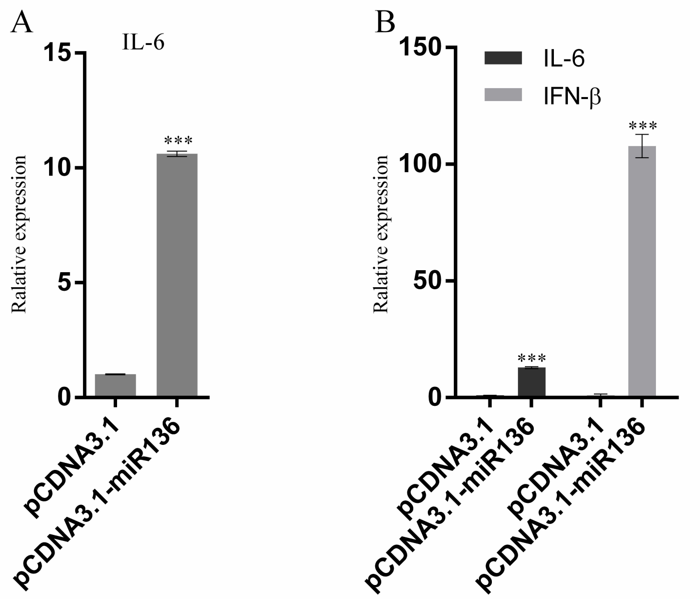

Supplementary Figure S4. Plasmid-expressed miR136 elevated both IL-6 and IFN-β expression in A549 cells. A549 cells were transfected with 1 μg pCDNA3.1-miR136 or empty vector. (A, B) IL-6 and IFN-β mRNA expression level was determined by RT-qPCR at 24 hours post-transfection. Bar graph data are presented as mean ± SD (n = 3). ***P < 0.001, as determined by performing a Student's t test.

Supplementary Figure S5

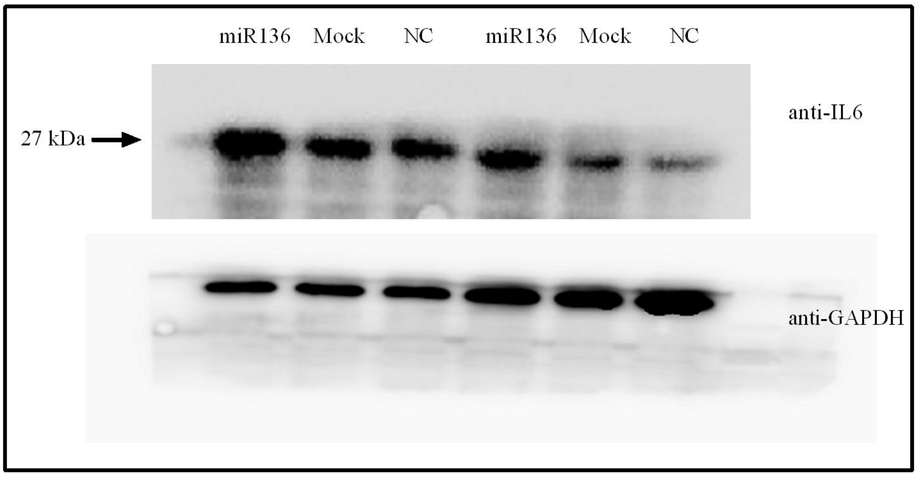

Supplementary Figure S6

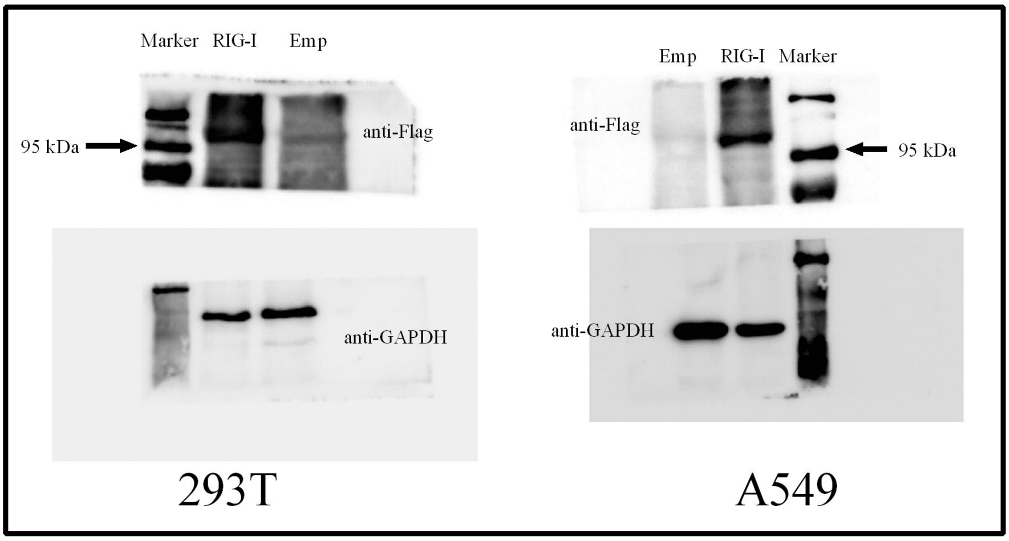

Supplementary Figure S7

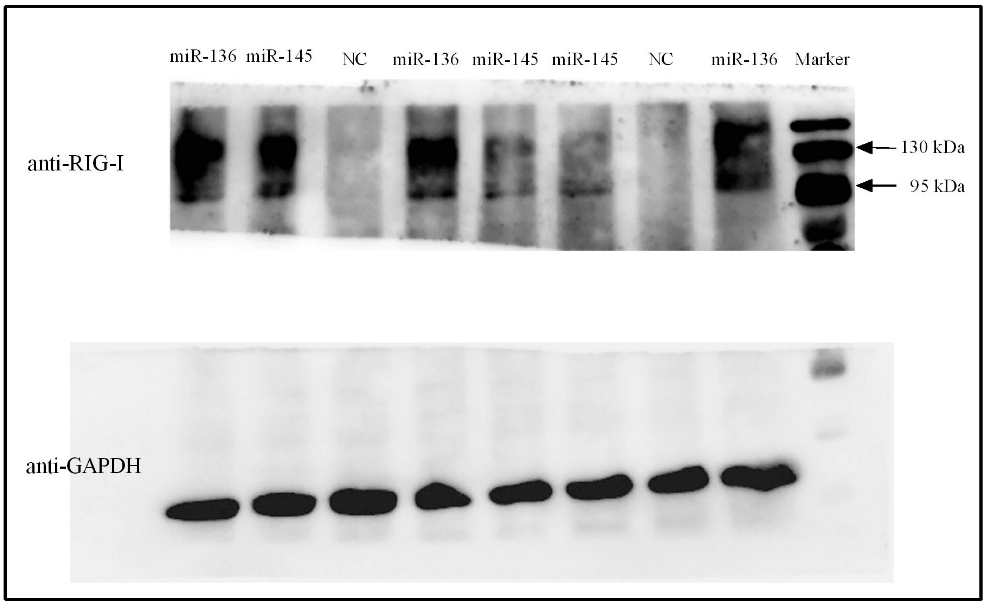

Supplementary Figure S8

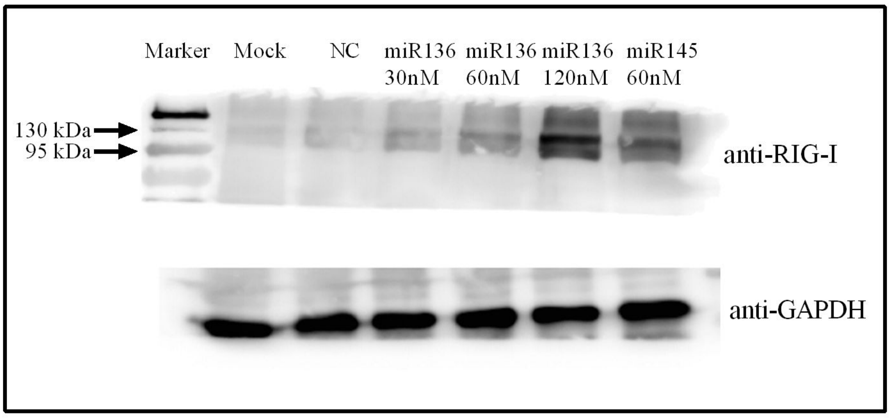

Supplementary Figure S9

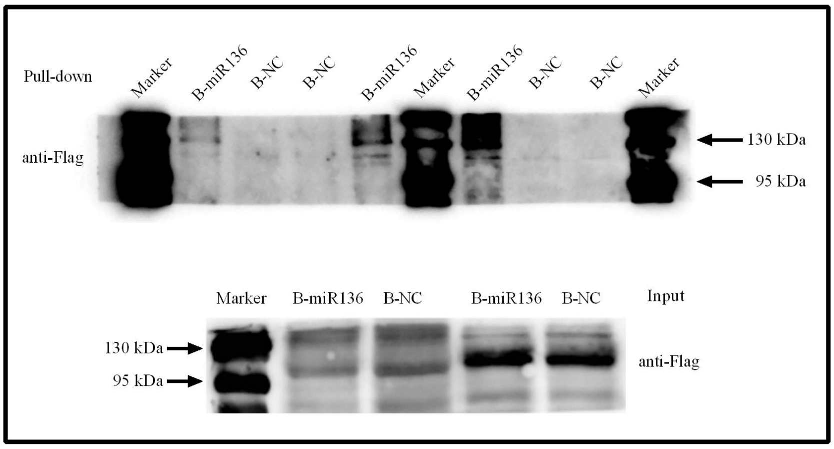

Supplementary Figure S10

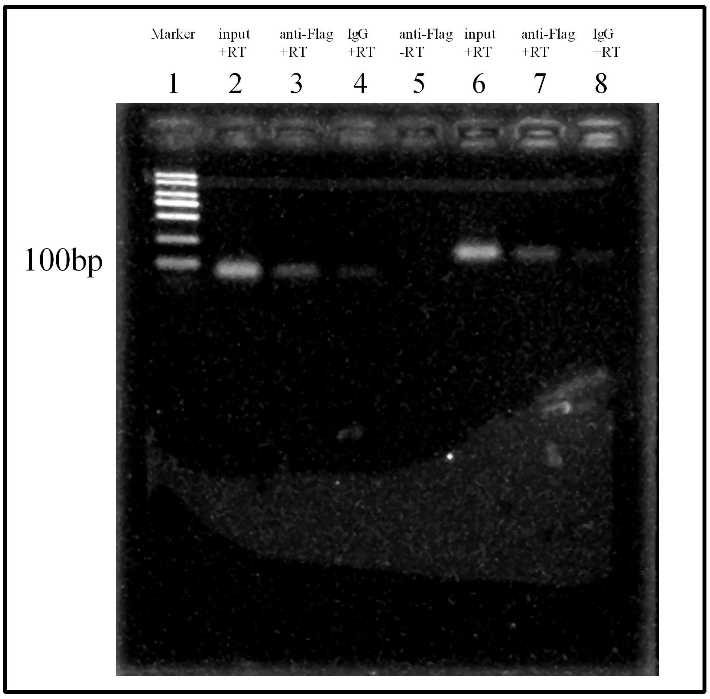

Supplementary Figure S10. Original gels of semiquantitative PCR. The molecular weight of putative PCR products was approximately 70 bp. The products in lane 5, 6, 7, and 8 were run delayed when loading the samples.

Supplementary Table 1 . List of miRNAs found to be differentially regulated upon H5N1/HM infection in microarray analysis.

**miRNA**	**Fold change**	**t-test**
hsa-miR-139-3p	3.98	0.031
hsa-miR-136	4.96	6.84726E-05
hsa-miR-17*	2.41	0.0013
hsa-miR-196a	1.67	0.0009
hsa-miR-340	0.46	0.034
hsa-miR-937	0.3	0.012
hsa-miR-129*	0.58	0.003

Supplementary Table 2. Primers, siRNA and miRNAs used in this study, bases in red were selectively 2′O-methyl modified in some experiments. siRIG-3 sequences, acquired from Williams BR[1], were used for RIG-I knockdown.

Name	Sequence (5'-3')
miR136 precursor-F	TAGAGATCTGATGGCTCCTCCATGTCTTGGAGTAGA
miR136 precursor-R	TAGTCTAGAGACCCTGATACTGCCACTTCACAAGAT
IL-63'UTR-F	TAGCTCGAGCATGGGCACCTCAGATTGTT
IL-63'UTR-R	TAGGTCGACGCTGAATTTTTTAAAATGCC
IL-63'UTRDeletion-F	GTCCACTGGGCACAGAACTAAAAGTATGAGCGT
IL-63'UTRDeletion-R	ACGCTCATACTTTTAGTTCTGTGCCCAGTGGAC
IL-6 3'UTR Mut-F	TTCTCTAGAGACAACTAAAAGTAT
IL-6 3'UTR Mut-R	ATACTTTTAGTTGTCTCTAGAGAA
IL6-F	AGGAGACTTGCCTGGTGAAA
IL6-R	CAGGGGTGGTTATTGCATCT
GAPDH-F	GCACCGTCAAGGCTGAGAAC
GAPDH-R	TGGTGAAGACGCCAGTGGA
IFN-β-F	GCTTGGATTCCTACAAAGAAGCA
IFN-β-R	ATAGATGGTCAATGCGGCGTC
OAS1-F	AGCTTCGTACTGAGTTCGCTC
OAS1-R	CCAGTCAACTGACCCAGGG
TNF-α-F	GAGGCCAAGCCCTGGTATG
TNF-α-R	CGGGCCGATTGATCTCAGC
IFN-α-F	GCCTCGCCCTTTGCTTTACT
IFN-α-R	CTGTGGGTCTCAGGGAGATCA
miR136-RT	GTCGTATCCAGTGCAGGGTCCGAGGTATTCGCACTGGATACGACTCCATC
U6-RT	GTCGTATCCAGTGCAGGGTCCGAGGTATTCGCACTGGATACGACAAAATA
miR136 Forward primer	CGCCACTCCATTTGTTTTGA
U6 Forward primer	CTCGCTTCGGCAGCACATA
Universal reverse primer	GTGCAGGGTCCGAGGT
hsa-mir-136 mimic forward	ACUCCAUUUGUUUUGAUGAUGGA
hsa-mir-136 mimic reverse	CAUCAUCAAAACAAAUGGAGUUU
hsa-mir-29a mimic forward	UAGCACCAUCUGAAAUCGGUUA
hsa-mir-29a mimic reverse	ACCGAUUUCAGAGAUGGUGCUAUU
hsa-mir-145 mimic forward	GUCCAGUUUUCCCAGGAAUCCCU
hsa-mir-145 mimic reverse	GGAUUCCUGGGAAAACUGGACTT
Negative Control mimic forward	UUCUCCGAACGUGUCACGUUU
Negative Control mimic reverse	ACGUGACACGUUCGGAGAAUU
siRIG-I sense	pGCCAGUGGAGAUCACAAUAUUCUga
siRIG-I antisense	UCAGAAUAUUGUGAUCUCCACUGGCUU
siRNA control forward	UUCUCCGAACGUGUCACGUUU
siRNA control reverse	ACGUGACACGUUCGGAGAAUU
miR-136 inhibitor	UCCAUCAUCAAAACAAAUGGAGU
miR-136 inhibitor negative control	CAGUACUUUUGUGUAGUACAA

Supplementary Table 3. Putative target genes of miR-136 (genes in red are closely related to IAV replication).

Gene Symbol	Genes identifier	miRanda	picTar	PITA	RNA22	Targetscan
ADIPOR2	79602	√	√		√	
AKAP11	11215	√				
AMOTL1	154810	√				
ATF2	1386	√				
BTBD3	22903	√			√	
C6orf62	81688	√				
CADM1	23705	√				
CALM2	805	√	√		√	
CLIC4	25932	√				
CTNNB1	1499	√				
DUSP16	80824	√		√		
DYRK1A	1859	√				
E2F1	1869	√		√		√
FOXJ3	22887	√			√	
GOLT1B	51026	√			√	
HOXC10	3226	√	√	√		√
IL-6	3569	√				
KLF4	9314	√				
KPNB1	3837	√				
KRAS	3845	√				
LCOR	84458	√		√		
MAP2K4	6416	√				
MAZ	4150	√				
MED12	9968	√				
MINK1	50488	√				
MSL2	55167	√	√	√		
MTPN	136319	√	√			
NFE2L1	4779	√				
NMT1	4836	√		√		
PAFAH1B1	5048	√		√		
PNRC2	55629	√				√
PPP1R14C	81706	√		√		
PURB	5814	√	√	√		√
RAB14	51552	√				
RAD23B	5887	√		√		√
RFX7	64864	√				
RGS16	6004	√		√		
RNF2	6045	√		√		
RPUSD4	84881	√				√
SFRS1	6426	√	√	√		√
SNAPC1	6617	√				√
TMBIM6	7009	√		√		
TMEM33	55161	√		√		

1. Marques, JT. et al. A structural basis for discriminating between self and nonself double-stranded RNAs in mammalian cells. *Nat Biotechnol*; **24**: 559-565. (2006)
